# Generational Differences in Perceptions of Food Health/Risk and Attitudes toward Organic Food and Game Meat: The Case of the COVID-19 Crisis in China

**DOI:** 10.3390/ijerph17093148

**Published:** 2020-04-30

**Authors:** Xiaoru Xie, Liman Huang, Jun (Justin) Li, Hong Zhu

**Affiliations:** 1 Division of Geography and Tourism, Department of Earth and Environmental Sciences, Katholieke Universiteit Leuven, 3000 Leuven, Belgium; xiaoru.xie@kuleuven.be; 2 School of Tourism Management, South China Normal University, Guangzhou 510006, China; Mandyhuang@m.scnu.edu.cn; 3 Southern Marine Science and Engineering Guangdong Laboratory, Zhuhai 519000, China; 4 School of Geographical Sciences, Guangzhou University, Guangzhou 510006, China

**Keywords:** health/risk perception, attitude, organic food, game meat, food safety crisis, China

## Abstract

In December 2019, a novel laboratory-confirmed coronavirus (2019-nCoV) infection, which has caused clusters of severe illnesses, was first reported in Wuhan, the capital of Hubei province, China. This foodborne illness, which reportedly most likely originated in a seafood market where wild animals are sold illegally, has transmitted among humans through close contact, across the world. The aim of this study is to explore health/risk perceptions of and attitudes toward healthy/risky food in the immediate context of food crisis. More specifically, by using the data collected from 1008 respondents in January 2020, the time when China was hit hard by the “Corona Virus Disease 2019” (COVID-19), this study investigates the overall and different generational respondents’ health/risk perceptions of and attitudes toward organic food and game meat. The results reveal that, firstly, based on their food health and risk perceptions of healthy and risky food, the respondents’ general attitudes are positive toward organic food but relatively negative toward game meat. Secondly, older generations have a more positive attitude and are more committed to organic food. Younger generations’ attitude toward game meat is more negative whereas older generations attach more importance to it because of its nutritional and medicinal values. In addition, this research also indicates that the COVID-19 crisis influences the respondents’ perceptions of and attitudes toward organic food and game meat consumption. However, the likelihood of its impact on older generations’ future change in diets is smaller, which implies that older generations’ food beliefs are more stable.

## 1. Introduction

Food choice and consumption are dynamic, situational, and complex, having resulted from the (non-)sensory characteristics of food and influenced by cultural and socio-affective factors as well as reliable information available about the food [1]. It is a daily activity that can result in both good and bad consequences for our bodies. Game consumption is complicated, interrelated with growth, prosperity, and consumption habits in economic, cultural, and social aspects. For example, with a growing market, game meat consumption has become a social status and fashionable lifestyle. The symbolic role of wildlife is obvious in China’s developed cities. It has become a symbol of elite status and fashionable lifestyle for some people to buy and eat wildlife animals. Despite being perceived as healthy based on its natural and nutritional characteristics, game meat consumption is also risky because of the potential microbiological contaminant. In December 2019, a foodborne illness, which was caused by a novel coronavirus (2019-nCoV) and reportedly most likely originated from the wild animal pangolin, was first identified in Wuhan, the capital of Hubei province, China [2,3]. It has spread rapidly through human-to-human contact in the whole of China and hit the society hard, across the world. The COVID-19 crisis has caused a food anxiety about game meat. Given the possible microbiological contaminants in game meat, strategies have been conducted in response to the emergence of the COVID-19 crisis to discourage the consumption of game meat. The Chinese government has temporarily banned the trade in wild animals, including domestic transport and sales in markets, restaurants, and through online platforms [4].

When the coronavirus crisis hit China’s farms and food supply chain, the public moved to a high-value organic food for its safer characteristics such as lower microbiological risks and chemical contaminants. As research has demonstrated, food anxiety and health scares caused by food safety crises can change consumers’ sensitivity and beliefs about food health, turn them to diets perceived as more natural, and make organic food more popular [5]. However, owing to variegated ways of defining and standardizing organic food, the boundary between organic food and game meat is not clear. The “natural,” “nutritional,” and “tasty” attributes of game meat lead to a common cognition that game meat is organic. Recent studies have revealed that game meat has high protein, low fat, and low calorie [6,7]. It can add a healthy alternative to the menus of meat lovers [1]. However, being the origin of foodborne crisis, game meat is also regarded as a risky food with microbiological hazards. It constitutes one of the unique sources of foodborne illness in humans [8]. Considering the microbiological safety issues, handling, and/or consumption of game meat might expose humans to diverse microorganisms and result in novel diseases [9,10].

Organic food and game meat consumption are complicated issues interrelated with different consumption habits. Generational differences in food consumption do exist as age can influence the individual’s attitudes and behavior and drive certain wants and needs [11]. Identifying a “generational effect” in organic food and game meat consumption is important because sharing historical and social life experiences of a generational cohort impacts the way people of that generation behave and distinguishes them from other generations [12]. As it comes of age, each generation experiences a fresh interaction with traditional values and principles [11] and is likely to develop distinct preferences, which distinguish their attitudes toward food. For example, the millennials, also known as Generation Y and mainly defined as the 1980s and 1990s generations, who grew up at a time of rapid economic and technological development [13], differ from previous generations in various ways. For example, they are characterized as being highly diverse, educated, and technology-natives, holding distinctive values and disparate views on food consumption [14]. As Turow argues [15], the younger generations are integrating food into their lives actively and purposefully, attaching more daily importance and value to it than any older generations. In addition, because of China’s dramatic and rapid transformations in economy, politics, and culture, the gaps between the new and older generations are arguably larger than in Western countries [16]. These arguments enlighten us to explore whether differences in organic food and game meat consumption are linked to generational differences.

This research aims to gain a general understanding of health/risk perceptions and attitudes toward healthy/risky food in the immediate context of food crisis as well as to understand the impact of generational difference on perceptions and attitudes. It provides an empirical survey to investigate the overall and different generational respondents’ health/risk perceptions and attitudes toward organic food and game meat during the outbreak of the COVID-19 crisis in China, utilizing data collected from 1008 respondents in January 2020, when the crisis hit the whole society.

## 2. Literature Review

### 2.1. Food Safety Studies

Food safety is one of the key areas of focus in public health. Food safety incorporates hazardous factors such as animal disease-related safety issues and microbiological safety, and represents consumers’ health concern regarding residues in food production, processing, and distribution. Triggered by nutritional hazards in food crises, food safety issues have received increasing attention in nutritional studies [17,18]. In this regard, it is considered a priority to implement food safety assurance at the farm level [19,20]. Organic food, generally believed to have higher nutritional content than conventionally grown food, such as higher vitamin C, higher dry matter, and lower nitrate content [21,22,23], is of growing interest due to food safety concerns.

Food safety is one of the main reasons for organic food purchasing [24]. Nowadays, the public is more concerned about food safety because of the extensive food scandals and intensively publicized debates on genetically modified organisms, which have led to increasing awareness and pursuit of safer alternatives [25,26]. Scared of food hazards, many consumers choose organic food to avoid risks. Consequently, further attention has been paid to the safety, nutrition, and health aspects of organic food [23].

The definitions and standards of organic food, however, vary according to diverse situations and contexts. Although food labeled as organic is commonly perceived to be healthier, tasty, and natural, without additional risk of food poisoning and free from contamination [27], the criteria are negotiated in the light of experience, practicalities, and moral considerations [28,29]. It is also claimed that the term “organic” refers to the situated production process instead of the product itself—the label is just a guarantee of a particular process [30,31,32]. For example, in addition to their nutritional qualities, African wildlife meats are considered organic for they are reared as free-range and meet the general criteria of organic production [33].

### 2.2. Game Consumption

Game consumption is a complicated issue interrelated with growth, prosperity, and consumption habits in economic, cultural, and social aspects. In keeping with population growth, increasing buyer power, and globalization, game trade, including skins, medicinal ingredients, and food has become a burgeoning business around the world [34,35]. It provides an income for some poor people and considerable revenue for the nation [35]. In Hainan, China, for instance, with the improvement in the town-dwellers’ standard of living, people have become more interested and able to add wildlife to their daily menu, which offers the local villagers a commercial way to increase their income [36].

Southeast Asia has been identified as a “wildlife trade hotspot” [35], where the consumption of wildlife for medical, superstitions, and nutritional reasons is culturally rooted [37]. Traditional Chinese medicines (TCMs), with some of the ingredients derived from wildlife, have become an integral part of Chinese culture [38]. The perceptions of therapeutic benefits of TCMs are based largely on long-standing beliefs dating back thousands of years [38,39]. Similarly, game meat in Cambodia for use in traditional medicines is a practice with deep historical roots [40].

In addition, with a growing market, game meat consumption has become a social status and fashionable lifestyle [39]. In Vietnam, for example, the majority of wild animals are now embedded in commercial networks to serve the growing urban middle class in provincial towns and cities [41]. The symbolic role of wildlife is also obvious in China’s developed cities, especially large cities in south China. It has become a symbol of elite status and fashionable lifestyle for some people to buy wildlife animals as crafts and souvenirs, eat game meats, and dress in animal furs [42].

### 2.3. Food Risk: Perception and Attitude

Risk is “the probability that the potential of a hazard will be realized” and “a combination of the probability with the harm itself” [43,44]. Before taking action to control, reduce, or eliminate risks, decisions must be made to estimate which risks are important and which can safely be ignored [44,45].

Food health and risk perception are two vital factors concerning the acceptance of food, and the two are inversely and causally related [46]. Risk associated with food can be presented in various forms ranging from microbiological risks, nutritional deficiencies, physical and chemical contaminants to processing, handling, and eating risks [44,47]. Contaminated food is risky since it poses threats to consumers’ health and might cause illness and even death [48,49].

Empirical research on food risk originated from debates on the genetic modification of food and considerable food scares in the 1990s, especially the mad cow disease (BSE) [50]. With the development of the psychometric paradigm, scholarly attention has been paid to the perception of food risk [50]. Owing to several cases of food safety issues, food safety risk perception, which is a perception of the potential risk in food safety, has gained substantial attention in recent years [49]. Miles and Frewer [51], for example, argue that food risk perception is multifaceted and results from risks associated with health, environment, economy, animals, and future generations. In terms of the socio-demographic characteristics, Nardi et al. [49] highlight that age can positively drive safety risk perception because it encourages consumers to think about life threats.

Food risk perception plays a significant role in attitudes [49]. Attitude is a psychological tendency that is expressed by evaluating things, the self, and others, involving the positive and negative aspects of being favorable or unfavorable, likable or unlikable, and good or bad [52,53]. It is “the predisposition toward a particular object, reflecting behavioral, normative, and control beliefs that are directly related to the consumer’s intention and, consequently, his or her behavior” [49]. Attitudes can be judgments, memories, or both and stable or temporary, having resulted from relatively long-term processes such as socialization as well as short-term exposure to information [52].

The construction of attitudes is often concerned with and reciprocally related to affects, beliefs, and (overt) behaviors that can serve as bases for attitudes [52,54]. Albarracín, Johnson, & Zanna [52] note that affect is the feeling that people experience; beliefs are cognitive thoughts about the perceived likelihood that an attribute is related to an object or event—the referent of a belief is a proposition and it can also refer to subjective experiences; and behaviors are conceptualized as the overt actions of an individual [55,56,57].

In terms of feeling, Clore & Schnall [58] argue that “decision-relevant feelings might come from vividly imagined consequences of a decision.” Affect is also associated with risk perception [59]. Sensory inputs can generate visceral reactions, which can automatically induce avoidance [52]. Emotions such as fear, guilt, and empathy are the strongest affective responses and fear, which is driven by uncertainty, appears more salient in food crises [60,61,62,63].

## 3. Methods

### 3.1. Data Collection and Analysis

This research is part of a bigger research project aimed to investigate mutual relations between organic food consumption, game meat consumption, and foodborne illness. The survey was conducted with a random sampling approach in January 2020. WeChat (the most used social media application in China) was employed to recruit questionnaire participants. The questionnaire was posted through a web-based link on WeChat. This link was generated through a free Chinese professional survey website *Wenjuanwang* (https://www.wenjuan.com/, a website like SurveyMonkey), which designed the online questionnaire (in Chinese). *Wenjuanwang* also enables researchers to establish, distribute, collect, and manage their questionnaires online.

Potential respondents could fill in the questionnaires through WeChat on their mobile phones, which accelerated the data collection during the outbreak of the COVID-19 crisis. A total of 1008 usable responses were collected for data analysis. Table 1 shows the socio-demographic profiles of the respondents. In China, the generation is always described as “the post-80s generation”, “the post-90s generation”, etc. To investigate age differences, the respondents were divided into four age groups: aged below 20, 20–29, 30–39, and 40 and above. Data analysis was carried out with the statistic software SPSS, computed as means, standard deviations, and F-tests of differences.

### 3.2. Questionnaire and Measurements

#### 3.2.1. Questionnaire

A structured four-section questionnaire was formulated based on a literature review involving perception, attitude, behavior, and intention of organic food and game meat consumption [1,7,8,10,64,65,66,67], as well as other projects conducted by the authors associated with research in organic food production and consumption. It included questions on the relation between game meat consumption and foodborne illness, perceptions of and attitudes toward organic food and game meat, and demographic profiles of the respondents. Only part of the questionnaire that is related to this research is presented here.

#### 3.3.2. Measurements

Health/risk perception of and attitudes toward organic food and game meat were measured by means of the items of four measurements: “organic” consciousness and attitudes toward organic food, health/risk perception and attitudes toward game meat, perceived change in future diet: stability of attitudes toward organic food, and perceived change in future diet: stability of attitudes toward game meat. The statements were rated on a five-point Likert scale.

***“Organic” consciousness and attitudes toward organic food*.** The respondents’ attitudes toward organic food was measured by their “organic” consciousness with questions about how they perceive organic food, in terms of both individual and societal cognitions. Items 1 to 4 (see Table 2) were used to measure consumers’ attitudes toward organic food associated with personal considerations, with questions about how they rate the overall liking, importance of organic food in a daily diet, necessity of shifting from traditional food to organic food, and how wise they think it is to buy organic food. The societal considerations of organic food were reified with items of environmental and animal ethics.

***Health/Risk perceptions and attitudes toward game meat*.** The respondents’ health/risk perception and attitudes toward game meat, including the healthy and risky attributes of game meat, were assessed on a five-point scale. The assessment ranged from the overall liking, importance of game meat in daily diet, and food safety risk to health benefits (see Table 3).

***Perceived change in future diet: stability of attitudes toward organic food.*** To measure the stability of attitudes toward organic food, respondents were asked to estimate their frequency of daily organic eating, future possibility of increasing organic food eating frequency, and extent of influence of the COVID-19 crisis on future organic food eating (see Table 4).

***Perceived change in future diet: stability of attitudes toward game meat.*** Questions about the frequency of daily game meat-eating, future possibility of increasing game meat-eating frequency, and extent of influence of the COVID-19 crisis on future game meat-eating were employed to measure the respondents’ stability of attitudes toward game meat (see Table 5).

## 4. Results: Effect of Age on Health/Risk Perceptions and Attitudes toward Organic Food and Game Meat

### 4.1. “Organic” Consciousness and Attitudes toward Organic Food

As Table 2 reveals, the respondents’ attitudes toward organic food as health belief differs significantly among the four groups aged below 20, 20–29, 30–39, and 40 and above. However, the items of the environmental and animal ethics of organic food are not statistically significant across age. All the mean scores are above 3, which means the respondents have positive consciousness about organic food. The respondents aged 40 and above scored the highest among all four groups on “the extent of organic food favor,” “the importance of organic food in a daily diet,” and “the necessity of changing traditional food to organic food,” while the respondents aged 20–29 scored the lowest on “the extent of organic food favor,” “the importance of organic food in daily diet,” and “buying organic food is wise.” In general, the respondents aged 40 and above have significantly more positive attitudes toward organic food than other age groups, while the 20–29 group shows the lowest perceptions.

### 4.2. Health/Risk Perceptions and Attitudes toward Game Meat

As shown in Table 3, the respondents’ attitudes toward game meat are significantly different among the four disparate generations. It indicates that the respondents have a relatively negative attitude toward game meat. However, the respondents aged 40 and above showed a comparatively more positive attitude toward game meat than the other three age groups which expressed similar levels of concern about game meat hazards. The 40 and above age group had the lowest perceptions of the food risk of game meat, which was reflected in items 3 to 5. They had a more positive attitude toward game meat because of its nutritious and medicinal values. The 20–29 age group had the lowest perception of the nutritional and medicinal values of game meats. In general, the younger generations’ attitudes toward game meat were more negative than that of the older ones.

### 4.3. Perceived Change in Future Diet: Stability of Attitudes toward Organic Food

As shown in Table 4, the respondents’ eating frequencies of organic food are significantly different among the four disparate generations. Respondents under the age of 20 had the highest frequency of eating organic food in daily life—above the midpoint of 3—while the 30–39 group showed the lowest frequency. Although the 40 and above age group respondents also ate less organic food in their current daily life, they showed the highest willingness to increase their organic food eating in the future. In general, the COVID-19 crisis has had a positive influence on future increase in organic food eating. However, its influence on the older generations is lesser than on the younger generations.

### 4.4. Perceived Change in Future Diet: Stability of Attitudes toward Game Meat

As shown in Table 5, the respondents’ eating frequency of game meat is relatively low in their daily diet, and they show a very negative attitude on the future increase in game meat consumption. However, the factor “the future possibility of increasing GM eating frequency” is not statistically significant across age groups. As for the influencing extent of the COVID-19 crisis to the future eating of game meat, Table 5 indicates that the crisis has an extremely significant impact. The younger generations, particularly the group aged 20 below, are significantly more likely to decrease the amount of game meat in their future diets than the older respondents. Thus, the COVID-19 crisis has a considerable negative influence on the future eating of game meat. However, its impact on the older generations is smaller than the younger ones.

## 5. Discussion

### 5.1. Perceptions of and Attitudes toward Organic Food

Attitudes toward organic food show that generally, respondents aged 40 and above attached more importance on organic food than the other age groups, while the 20–29 age group respondents showed the lowest perceptions. This result is both consistent and contrary to some previous research. From the perspective of the influence of age on organic food consumption, research has been conducted within diverse situated contexts with different results. Rimal, Moon, & Balasubramanian [68], for example, observed that older respondents are less likely to buy organic food than younger ones. On the contrary, Geen and Firth [69] concluded that committed consumers of organic food tend to be older than the average population in the UK [67].

However, despite the older respondents attaching more importance on organic food, the younger generations have a higher frequency of daily eating, especially the respondents aged under 20. This interesting result might be explained by the fact that in China, organic food consumption is still in the infant stage. Although the amount of organic food consumption is increasing, the total quantity and frequency of organic food eating is still at a low level across the whole of China [70]. Parents who consider their children’s health constitute the main consumers of organic food, and because of the previous “one-child” policy introduced in 1980, most of them have only one child. However, as the authors were told in another research project by an organic farmer, because of economical consideration, some parents purchase organic food just for their children and seldom eat it themselves.

Therefore, the behaviors of “eating” and “buying,” which both mean “consumption,” might be different when the intention of “consumption” is considered. This might also be, at least in part, the reason why the 40 and above age group generally attached more importance on organic food, while the respondents who ate more organic food tended to be younger, especially under 20. Further research needs to verify this and investigate other possible explanations with more empirical examinations. This also implies that organic food perceptions of the older generations are based more on health characteristics. This implication is consistent with some previous research [71,72,73]. Healthfulness is a perceived short-term benefit of organic food [64]. The elders’ egoistic motives (health considerations) seem to come first rather than altruistic motives (ethical considerations).

In the future, the older generations might become more committed to organic food. They appeared more willing to increase the proportion of organic food in their future diets. Except for the considerations of their children’s health, and, in this research, the impact of the COVID-19 crisis, another explanation might lie in their own bodily needs. As noted by Yiridoe, Bonti-Ankomah, & Martin [74], consumers’ preference for organic food is largely influenced by their concerns for health and safety, which is in line with their perceived deterioration in human health over time; therefore, it motivates them to purchase organic food as insurance and/or investment in health. As Cullere & Dalle Zotte [75] put it, health acts as a strong motivation for consumers to change eating habits. However, situated in a particular context, a previous research on the willingness to pay for the organic/green food in China shows a contrary result. It found that younger people are more willing to pay more as the older people are not willing to change their eating habits and pay a premium price for organic food [76].

### 5.2. Perceptions of and Attitudes toward Game Meat

Attitudes toward game meat indicate that the respondents have a relatively negative attitude toward game meat consumption, which signifies game meat as risky food. Their negative attitudes are caused by perceptions of its food safety risk, as reflected in their high scores—a mean score of above 4.3—on the statement “eating game meat is of health hazard” (see Table 3). Their negative attitudes might also be enhanced by their anxiety during the severe outbreak of the COVID-19 crisis. As research in the UK and European countries reveals, food panics generally spur initial disruption of attitudes and behavior but are also “followed by a gradual return to the previous consumption equilibrium” [77]. This implies that the food crisis has an immediate effect on consumers. When situated in the particular context of the food crisis, humans’ food anxiety is enhanced.

On the other hand, respondents also attach game meat with somewhat “healthy” meanings, so they still sometimes “venture” to eat game meat, which might result from their perception of the nutritional and medicinal values of game meat. They are generally aware of the hazards of game meat. However, awareness does not necessarily indicate absolute avoidance. As Martins and Pliner [78] argue, “humans must balance the necessity of obtaining a wide variety of nutrients (typically, through the ingestion of numerous food sources) with the perils accompanying the ingestion of unfamiliar edibles (i.e., the possibility that these foods may be harmful or toxic).” Some studies also reveal that despite game meat being perceived as a health hazard with potential microorganisms and toxins and its consumption being far less than that of usual meat such as beef, pork, and fish, it still offers consumers a healthy meat alternative because of its nutritional value such as high protein, low cholesterol and fat, and an optimal ratio of unsaturated to saturated fatty acids [1,7,79,80]. Tomasevic et al. [7], for example, conducted a study on perceptions of different game meat in 10 European countries and pointed out that game meat is regarded as healthy and more organic than other types of meat.

Although being negative, the older respondents seem to have a significantly higher perception of game meat than the younger generations, which might be based more on its “health” benefits—being nutritious and medicinal—and they practice more “risky” behaviors. In game meat consumption, the eating frequency trend related to age is consistent with the trend of perceived risks. The older respondents eat more and have a lower risk perception of game meat than the younger respondents. This is partly consistent with previous research indicating that age has an impact on how respondents perceive health and food safety risks, but older respondents are more likely to have a higher perception of health and food safety issues as risks than younger respondents [81].

The reason might be that when balancing the benefit and risk of game meat, the older respondents in this research attached more importance to the “medicinal” attributes of game meat, which could also be reflected in their higher scores on game meat’s medicinal values. In China, except for its nutritional value, game meat is also perceived to have high medicinal value—as Li et al. (2020) note [82], some consumers hold the belief that game meat has certain therapeutic effects. In addition, another belief rooted in the game meat consumers’ mind might derive from the philosophy of medicine food homology, which is misunderstood as that “one is made of the supplements they eat” [82]. Considering the age difference, these cognitions are engrained more in the elders’ minds.

### 5.3. The Impact of the COVID-19 Crisis

The impact of the COVID-19 crisis on respondents’ perceptions of health and risk is significant and would lead to the respondents’ perceived future increase in the consumption of organic food and decrease in game meat consumption. This is in line with some studies revealing that food anxiety and health scares caused by food safety crises can change consumers’ sensitivity and belief about food health, which turns them to diets perceived as more natural and makes organic food more popular [5,77,83,84]. For instance, the discovery of mad cow disease (BSE) in Canada and the US generated consumer scares about the safety of meat, besides rising the demand for naturally raised and organic meat [77,85].

In addition, the perceived extent of influence of the COVID-19 crisis on future change in organic food and game meat-eating shows that the older generations’ food belief is more stable. As this research reveals, the influences of the COVID-19 crisis on future change in diets, both organic food and game meat, is smaller on the older than on the younger generations. The older generations have a more fixed view on food health and risk.

## 6. Conclusions

This research aims to explore health/risk perceptions and attitudes toward healthy/risky food in the immediate context of food crisis, as well as to understand different generations’ attitudes toward it. Our findings reveal that a statistically significant “generational effect” is found in healthy and risky food consumption. The older generations have a more positive attitude toward organic food, and they have a higher willingness to increase their future eating frequency, which means they would be more committed to eating organic food than would the younger generations. The younger respondents have a more negative attitude toward game meat. The older generations attach more importance on game meat because of its healthy values of being nutritious and medicinal. As people become more affluent, they are able to choose organic food since it has more beneficial nutrients. Thus, eating organic food is moving up more and more consumers’ agenda. Promoting production and trading opportunities for the certified organic food to meet sustainability goals should be a top priority of local government in the developing countries. This research maps a positive future for the organic food market, which might encourage more farmers to involve in organic agriculture.

The COVID-19 crisis has a positive impact on the respondents’ attitude toward organic food and negative impact on their attitude toward game meat, which implies that food anxiety and health scares caused by food safety crises can change consumers’ sensitivity and belief about food health and risk. The impact of the food crisis on the older generations’ future change in diets is smaller, which means that the older generations’ food belief is more stable. Based on food health, they have a more solid belief on food than do the younger respondents. However, the COVID-19 epidemic has created some opportunities for positive change in China. For example, the government prepares a ban on trade of wildlife to improve public awareness on the health risks of the consumption of non-aquatic wild animals for food. On the supply-side, incentives can be created to encourage more investment on building a healthier food system. In addition, this research also shows a positive future for organic food consumption, which might encourage more farmers to engage in organic food production.

## 7. Limitations and Future Research Suggestions

This study has a few limitations. Firstly, the items used to measure the constructs in this study may not be the best representation for assessing the construct of interest. Secondly, the use of internet to aid research practice has become more popular. However, depending on the sampling method, the information collection may not be more accurate compared to offline surveys. Thirdly, future research might consider empirically testing other factors that explain why the 40 and above age group generally attached more importance on organic food.

## Figures and Tables

**Table 1:** Socio-demographic profiles of the respondents.

Variable	N	%
Gender		
Male	295	29.3
Female	713	70.7
Age		
Below 20	206	20.4
20–29	476	47.2
30–39	125	12.4
40 and above	201	19.9
Education		
Elementary school	3	0.3
Middle school	11	1.1
High school	34	3.4
Junior college degree	303	30.1
Bachelor	429	42.6
Master or PhD	228	22.6
Income		
5000 yuan or less	643	63.8
5001–10,000 yuan	212	21.0
10,001–25,000 yuan	112	11.1
25,001–50,000 yuan	28	2.8
More than 50,000 yuan	13	1.3

**Table 2:** The means, standard deviations, and F-tests of differences of the measurement “‘organic’ consciousness and attitudes toward organic food” across age.

“Organic” Consciousness and Attitudes toward Organic Food										
Items	Below 20 (N = 206)		20–29 (N = 476)		30–39 (N = 125)		40 and above (N = 201)		F	Sig.
	Mean	SD	Mean	SD	Mean	SD	Mean	SD		
(1) the extent of OF favor (1 = not at all; 5 = very much)	3.70	0.886	3.63 ^a^	0.767	3.89	0.863	3.98 ^b^	0.728	10.752	0.000 *
(2) the importance of OF in daily diet (1 = not at all; 5 = very important)	3.82	0.799	3.69 ^a^	0.766	3.75	0.973	3.93 ^b^	0.784	4.593	0.003 *
(3) the necessity of changing traditional food to OF (1 = not at all; 5 = very necessary)	3.02 ^a^	0.891	3.03	0.792	3.25	0.989	3.48 ^b^	0.878	15.488	0.000 *
(4) buying OF is wise (1 = not at all; 5 = very wise)	3.84 ^b^	0.793	3.64 ^a^	0.747	3.81	0.84	3.78	0.763	4.255	0.005 *
(5) OF is beneficial for environmental sustainability (1 = strongly disagree; 5 = strongly agree)	3.92 ^b^	0.829	3.85 ^a^	0.775	3.94	0.883	3.87	0.764	0.714	0.544
(6) OF is beneficial for animal welfare (1 = strongly disagree; 5 = strongly agree)	3.87 ^b^	0.86	3.82	0.752	3.94	0.901	3.81 ^a^	0.809	0.969	0.406

(“OF” as organic food; * Significant at a 0.05 level; Superscript “a” indicates the lowest score; Superscript “b” indicates the highest score).

**Table 3:** The means, standard deviations, and F-tests of differences of the measurement “health/risk perceptions and attitudes toward game meat” across age.

Health/Risk Perceptions and Attitudes toward Game Meat										
Items	Below 20 (N = 206)		20–29 (N = 476)		30–39 (N = 125)		40 and above (N = 201)		F	Sig.
	Mean	SD	Mean	SD	Mean	SD	Mean	SD		
(1) the extent of GM favor (1 = not at all; 5 = very much)	1.55 ^a^	0.749	1.62	0.803	1.88	0.829	2.16 ^b^	0.926	26.169	0.000 *
(2) the importance of GM in daily diet (1 = not at all; 5 = very important)	1.58 ^a^	0.785	1.66	0.787	1.62	0.758	1.83 ^b^	0.717	3.888	0.009 *
(3) eating GM is of health hazard (1 = strongly disagree; 5 = strongly agree)	4.56	0.701	4.55	0.701	4.58 ^b^	0.599	4.31 ^a^	0.703	7.109	0.000 *
(4) GM has high nutritional values (1 = strongly disagree; 5 = strongly agree)	2.22	1.054	2.17 ^a^	0.945	2.34	0.934	2.60 ^b^	0.837	10.471	0.000 *
(5) GM has high medicinal values (1 = strongly disagree; 5 = strongly agree)	2.42	1.100	2.35 ^a^	1.006	2.57	0.962	2.68 ^b^	0.831	5.698	0.001 *

(“GM” as game meat; * Significant at a 0.05 level; Superscript “a” indicates the lowest score; Superscript “b” indicates the highest score).

**Table 4:** The means, standard deviations, and F-tests of differences of the measurement “perceived change in future diet: stability of attitudes toward organic food” across age.

Perceived Change in Future Diet: Stability of Attitudes toward Organic Food										
Items	Below 20 (N = 206)		20–29 (N = 476)		30–39 (N = 125)		40 and above (N = 201)		F	Sig.
	Mean	SD	Mean	SD	Mean	SD	Mean	SD		
(1) the frequency of daily OF eating (1 = never; 5 = very frequent)	3.10 ^a^	0.867	2.86	0.819	2.72 ^b^	0.809	2.76	0.772	7.991	0.000 *
(2) the future possibility of increasing OF eating frequency (1 = must be; 5 = not at all)	3.73	0.897	3.64 ^b^	0.815	3.84	0.846	3.92 ^a^	0.865	5.746	0.001 *
(3) the influencing extent of the COVID-19 crisis to future increase OF eating (1 = not at all; 5 = very much)	3.69 ^b^	0.873	3.42	0.902	3.26 ^a^	1.069	3.34	0.978	7.275	0.000 *

(“OF” as organic food; * Significant at a 0.05 level; Superscript “a” indicates the lowest score; Superscript “b” indicates the highest score).

**Table 5:** The means, standard deviations, and F-tests of differences of the measurement “perceived change in future diet: stability of attitudes toward game meat” across age.

Perceived Change in Future Diet: Stability of Attitudes toward Game Meat										
Items	Below 20 (N = 206)		20–29 (N = 476)		30–39 (N = 125)		40 and above (N = 201)		F	Sig.
	Mean	SD	Mean	SD	Mean	SD	Mean	SD		
(1) the frequency of daily GM eating (1 = never; 5 = very frequent)	1.23 ^a^	0.495	1.28	0.601	1.47	0.532	1.5 ^b^	0.567	12.081	0.000 *
(2) the future possibility of increasing GM eating frequency (1 = not at all; 5 = must be)	1.24 ^a^	0.655	1.25	0.602	1.26	0.646	1.29 ^b^	0.59	0.324	0.808
(3) the influencing extent of the COVID-19 crisis to future decrease of GM eating (1 = not at all; 5 = very much)	4.43 ^b^	0.999	4.32	1.085	4.14	1.194	3.99 ^a^	1.158	6.696	0.000 *

(“GM” as game meat; * Significant at a 0.05 level; Superscript “a” indicates the lowest score; Superscript “b” indicates the highest score).
